# Supplementary material for: The causal relationship between the human gut microbiota and pyogenic arthritis: a Mendelian randomization study
Source: Front Cell Infect Microbiol. 2024 Nov 26;14:1452480. doi: 10.3389/fcimb.2024.1452480 (PMC11629706; doi:10.3389/fcimb.2024.1452480)
Supplement: Supplementary file 1 [file DataSheet1.zip › mendelian test/ebi-a-GCST90027556.csv_leave_one_out.pdf]

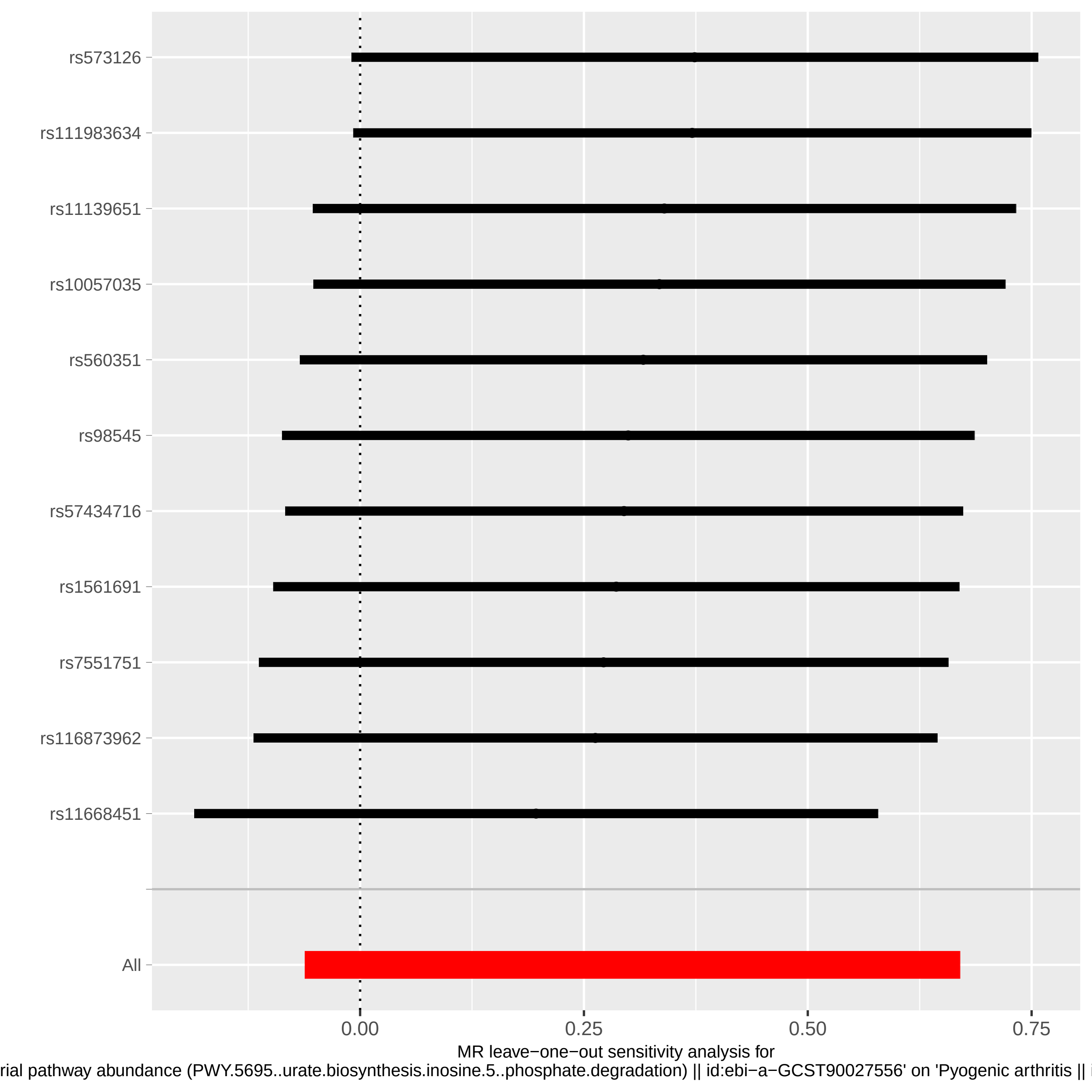
rs573126
rs111983634
rs11139651
rs10057035
rs560351
rs98545
rs57434716
rs1561691
rs7551751
rs116873962
rs11668451
All
0.00
0.25
0.50
0.75
MR leave−one−out sensitivity analysis for
rial pathway abundance (PWY.5695..urate.biosynthesis.inosine.5..phosphate.degradation) || id:ebi−a−GCST90027556' on 'Pyogenic arthritis || i
